# Evaluation of perfusion-driven cell seeding of small diameter engineered tissue vascular grafts with a custom-designed seed-and-culture bioreactor

**DOI:** 10.1371/journal.pone.0269499

**Published:** 2022-06-16

**Authors:** Sarah K. Saunders, Sam Y. Cole, Valeria Acuna Sierra, Johane H. Bracamonte, Stefano Toldo, Joao S. Soares

**Affiliations:** 1 Department of Mechanical and Nuclear Engineering, College of Engineering, Virginia Commonwealth University, Richmond, Virginia, United States of America; 2 Department of Biomedical Engineering, College of Engineering, Virginia Commonwealth University, Richmond, Virginia, United States of America; 3 Department of Internal Medicine, School of Medicine, Virginia Commonwealth University, Richmond, Virginia, United States of America; Georgia Institute of Technology and Emory University, UNITED STATES

## Abstract

Tissue engineering commonly entails combining autologous cell sources with biocompatible scaffolds for the replacement of damaged tissues in the body. Scaffolds provide functional support while also providing an ideal environment for the growth of new tissues until host integration is complete. To expedite tissue development, cells need to be distributed evenly within the scaffold. For scaffolds with a small diameter tubular geometry, like those used for vascular tissue engineering, seeding cells evenly along the luminal surface can be especially challenging. Perfusion-based cell seeding methods have been shown to promote increased uniformity in initial cell distribution onto porous scaffolds for a variety of tissue engineering applications. We investigate the seeding efficiency of a custom-designed perfusion-based seed-and-culture bioreactor through comparisons to a static injection counterpart method and a more traditional drip seeding method. Murine vascular smooth muscle cells were seeded onto porous tubular electrospun polycaprolactone scaffolds, 2 mm in diameter and 30 mm in length, using the three methods, and allowed to rest for 24 hours. Once harvested, scaffolds were evaluated longitudinally and circumferentially to assess the presence of viable cells using alamarBlue and live/dead cell assays and their distribution with immunohistochemistry and scanning electron microscopy. On average, bioreactor-mediated perfusion seeding achieved 35% more luminal surface coverage when compared to static methods. Viability assessment demonstrated that the total number of viable cells achieved across methods was comparable with slight advantage to the bioreactor-mediated perfusion-seeding method. The method described is a simple, low-cost method to consistently obtain even distribution of seeded cells onto the luminal surfaces of small diameter tubular scaffolds.

## 1. Introduction

Cardiovascular disease (CVD) is the number one cause of death worldwide, and those who are afflicted typically require several vascular reconstructive surgeries over the course of their life time [1]. The current gold standard in vascular reconstruction is the use of autologous vessels. The practice of using autologous grafts is risky as a separate surgery must be conducted to harvest the candidate vessel, effectively doubling the likelihood of infection and surgical complications. Additionally, patients may no longer have viable candidate vessels to be employed if their CVD is advanced. In small diameter applications, the use of synthetic grafts is limited due to complications with thrombosis, and decellularized xenografts typically illicit a pathogenic immune response [2]. Due to the limited number of viable alternatives, there is a defined need for engineered tissue vascular substitutes that integrate with host tissue to maintain patency comparable to that of autologous grafts. This is particularly significant for pediatric applications, when limiting the number of recurring surgeries over the lifetime of the patient is of key consideration [3]. Still, a cost- and time-effective method of consistently developing such engineered tissue vascular grafts (ETVGs) has yet to be elucidated [4, 5].

Tissue engineering has revolutionized the world of medicine by promising the creation of viable and functional replacement tissues via the utilization of scaffolds and autologous cells. Progress depends on the creation of increasingly complex engineered tissues aimed to perform distinct roles in treating a diverse range of diseases [6]. Scaffolds play an essential part in providing a beneficial microenvironment for cells, organizing the architecture of the developing engineered tissue, and aiding in the proper function of implants during host integration. Scaffolds for more elaborate tissues tend to have unique geometries and microstructures that require inventive methods to seed uniformly and efficiently. Specifically, tubular scaffolds of small diameter and significant lengths are essential for a variety of cardiovascular applications.

The method of cell seeding onto any scaffold determines the number of cells initially present for in vitro culture and their spatial distribution, which in turn dictates the proliferation, migration, and phenotype of cells as neo-tissue develops [7]. Achieving an even distribution of viable cells can prove challenging as the methods currently utilized are inherently inconsistent. Various techniques have been investigated to seed small diameter tubular scaffolds due to the unique problems associated with this class of geometries [8]. The most widely employed is dripping cell suspension onto the scaffold surface. However, this method is unreliable due to the manual nature of the procedure, and user independent consistency is challenging to achieve [9]. Various uncommon and less well-established cell seeding techniques that aim to use specific directing forces to control the seeding outcomes of the constructs are too hampered by the complexity of devices and procedures to be considered for regular use [10, 11]. Perfusion-based cell seeding, also called vacuum cell seeding or filtration cell seeding, has been employed in many applications such as seeding bone grafts, vascular grafts, heart valves, and other scaffolds of unique geometries. Perfusion based seeding has consistently been shown to improve cell adhesion to surfaces, increase proliferation, and encourage uniform cell distribution compared to traditional static methods [12–15].

Herein, we investigate perfusion seeding as a preferred method for luminal seeding of scaffolds with tubular geometries and quantify the performance of a custom-built perfusion seed-and-culture bioreactor against static seeding methods of cell delivery in regard to spatial distribution of seeded cells and their viability. The seed-and-culture bioreactor design was developed as a controlled environment capable of seeding and culturing 4 ETVGs simultaneously under identical conditions. Using the bioreactor system, a variety of environmental stimuli including dual axial mechanical stimulation and the addition of environmental growth factors may be administered to the developing ETVGs semi automatically with minimal handling. Having multiple directly comparable data points is essential to increasing the speed at which research may be conducted, but scaling up these experimental processes can be difficult. We suggest bioreactor-mediated perfusion seeding as a simple method that can be easily and reproducibly applied to 4 scaffolds concurrently in a bioreactor environment. Furthermore, the automatic process reduces variability in the seeding method, further reducing variability in ETVG quality between samples.

## 2. Methods

### 2.1 Scaffold fabrication

Polycaprolactone (PCL; Mn = 80,000), N,N-Dimethylformamide (DMF) and Tetrahydrofuran (THF) at high performance liquid chromatography grade were obtained from Sigma-Aldrich (St. Louis, MO) and used without further purification. The 14 wt% polymer solution was prepared by dissolving PCL in a solution of THF and DMF with a weight/weight ratio of 1:1 and stirred for 24 hours at 40°C. The solution was observed to be clear and without trapped air before every use.

The PCL solution was electrospun from a 1 ml syringe with a 26-gauge stainless steel blunt tip needle and a mass flow rate of 1.2 ml/h. The 2mm diameter brass mandrel was wrapped in aluminum foil and mounted 20 cm away from the needle on a stage set to translate 5 cm continuously at 1 cm/s as the mandrel rotates at 720 rpm. A high voltage (15 kV) was applied to the base of the needle for 35 minutes.

Each spin produced a PCL tubular scaffold with 2 mm inner diameter, 4 cm length, and with thickness ranging from 180–220 um which were cut from the aluminum collector sleeve and the sleeve removed. Scaffolds were sterilized via submersion in 70% ethanol overnight. A total of 52 electrospun PCL scaffolds were used in these experiments.

### 2.2 Bioreactor design

**Fig 1:**
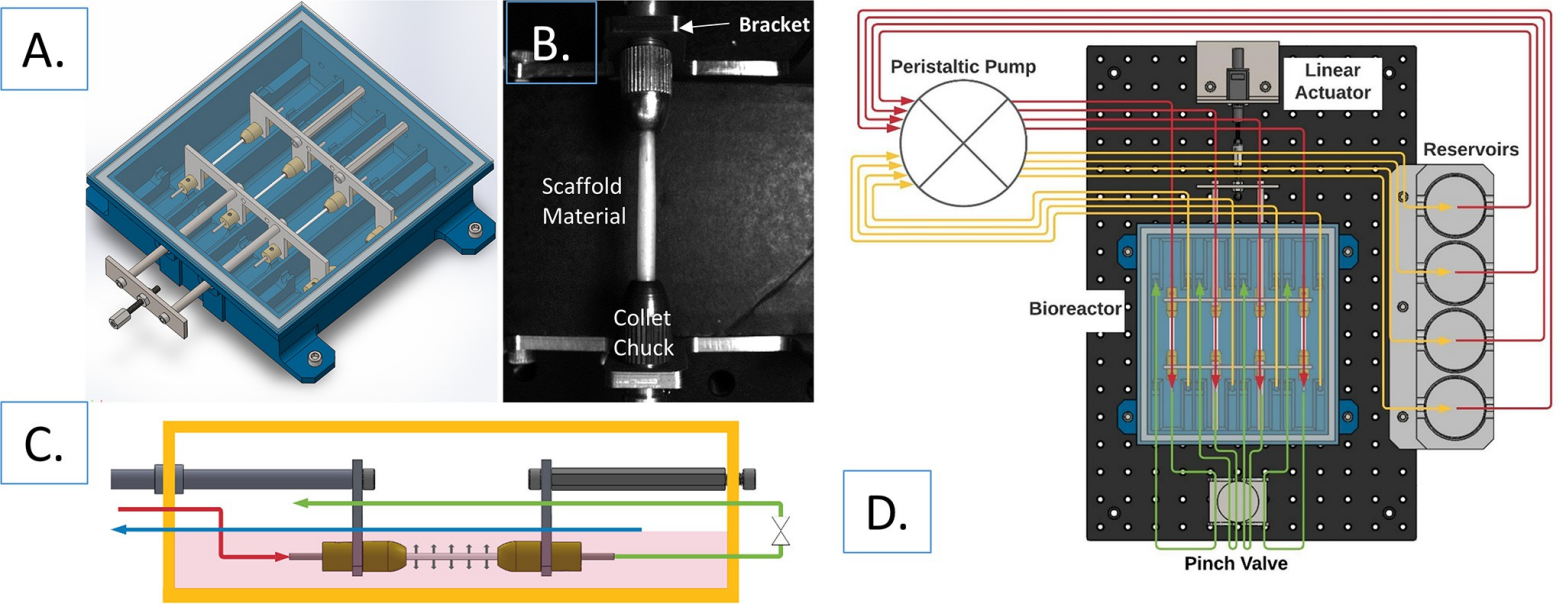
Bioreactor design. (A) The fully assembled bioreactor chamber. Intake and outtake tube holders are found on each side of the individual ETVG chambers. (B) An electrospun PCL tubular scaffold mounted with collet chucks in a four-pronged stainless-steel bracket. The 2mm cannulas affixed to either end of the scaffold form a tight seal with the tightened collet chucks. This connection makes tensile stretch and luminal pressurization possible while also leaving the lumen of the ETVG in direct contact with media flow. (C) Flow diagram within the bioreactor chamber. The red arrow shows media traveling from a reservoir to the ETVG lumen, the green arrow shows media movement out of the ETVG, out of the bioreactor, through the pinch valve, and back into the bioreactor. The blue arrow represents media being drawn from the bioreactor chamber to return to the reservoir. (D) Full schematic of bioreactor flow loop and intended features. Color coded as in panel C.

The bioreactor chamber (Fig 1A) was 3-D printed (Stratasys F170, Eden Prairie, MN) with a 13% infill using acrylonitrile butadiene styrene (ABS) with water-soluble QSR support material. The 3-D printed bioreactor chamber was coated with XTC-3D high performance epoxy resin, (Smooth-On, East Texas, PA) to prevent fluid leakage. The bioreactor was designed to seed-and-culture four tubular scaffolds in parallel channels (175 mm x 38 mm x 25 mm). The tubular scaffolds were fitted onto 2mm outer diameter stainless steel cannulas on each end such that 30mm of the graft length was available for seeding. Scaffolds were secured to the cannulas with parafilm, and mounted into two 4 pronged brackets with collet chucks (Fig 1B). The brackets are fastened at a fixed distance apart keeping the 30 mm length of the scaffolds straight and unstressed. Once assembled, the mounting bracket was placed in the bioreactor chamber and the scaffolds connected to their individual flow channels through the cannulas.

After assembly, the proximal end of the scaffold flow channels are connected to syringes, and the drain tubes located to the left of each proximal scaffold flow channel are sealed. The syringes are then used to rinse the mounted scaffolds once with 70% ethanol, 3x with sterile phosphate buffered saline, and then 2x with full culture medium containing 10% FBS and geneticin. The scaffolds are soaked in this solution overnight to prepare for the seeding step.

After the seeding period, the 4 ETVGs within the bioreactor are intended to be switched into culture mode under static or dynamic conditions (Fig 1C and 1D). Briefly, the syringes used for seeding are removed as well as the seals placed on the drainage tubes. These are then connected into the full flow loop which has been primed with full culture medium and the clamps at the distal end of the scaffold flow channels are removed. Culture medium is drawn from a media reservoir with a peristaltic pump, and pushed through the ETVG lumen, and returned to the ETVG chamber (Fig 1C). For fluid level balancing in each ETVG system, an additional flow line draws media from the bioreactor chamber back to the media reservoir. The intake and outtake tubes are located at opposite ends of the ETVG chamber, facilitating gentle media flow around the outer surface of the scaffold. For future dynamic culture, one bracket is connected to an actuator to impose identical axial stretch between all four ETVGs (Fig 1D). Downstream of the mounted ETVGs, a 4-channel pinch valve is used to impose circumferential stretch by timed inflation. These features are not directly used in this study; however, they are key aspects that were taken into consideration when designing the described seeding method.

All bioreactor components other than the chamber itself can be sterilized via steam autoclave for 30 min at 121 C. The bioreactor chamber is sterilized via immersion in 70% ethanol followed by UV sterilization for 1 hr. The bioreactor system was assembled aseptically in a laminar flow hood. The assembly was rinsed with PBS washes 3x prior to seeding.

### 2.3 Seeding experiment

**Fig 2:**
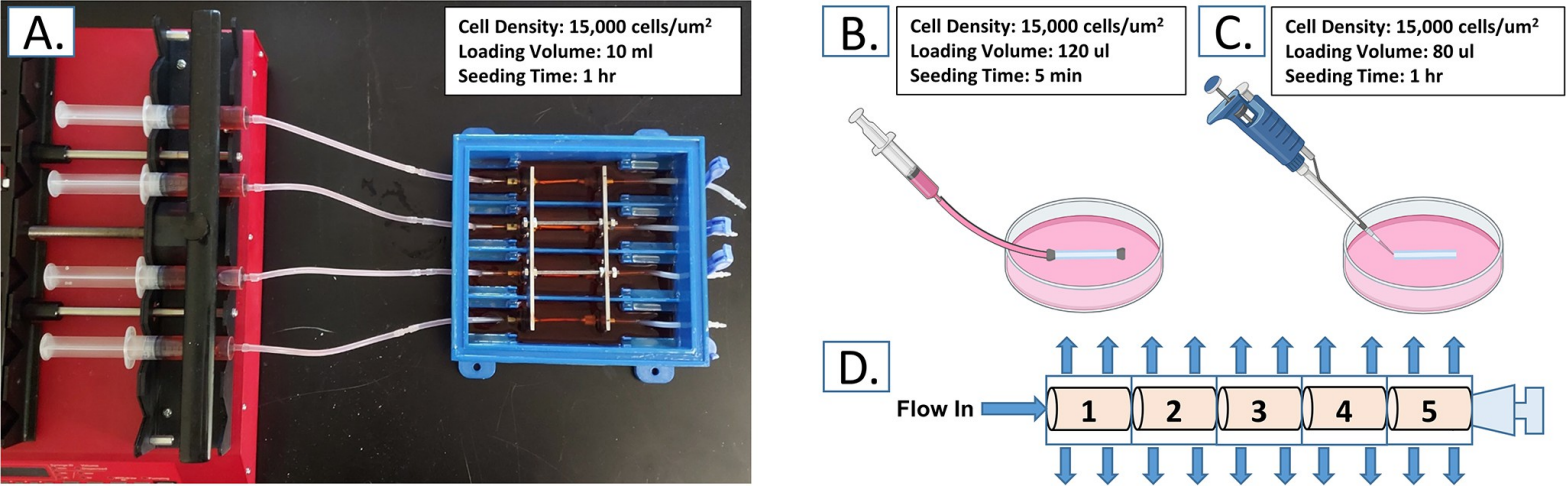
Seeding methodology. Seeding method diagrams for (A) the bioreactor mediated perfusion seeding method, (B) static injection counterpart seeding method and (C) drip seeding. (D) Operating principle of bioreactor mediated perfusion seeding method. Numbers indicate sample organization for analysis.

Murine vascular smooth muscle SV40LT-SMC cells (VSMCs) (ATCC; Manassas, VA, USA) were cultured in full growth medium containing Dulbecco Modified Eagle’s Medium (DMEM), 10% fetal bovine serum (FBS) and 200 mcg/mL geneticin (G418), all produced by Gibco. At passage 6, cells were harvested for seeding. Prior to seeding, all scaffolds were prewetted with full growth medium overnight. During and after seeding, scaffolds were maintained in a 5% CO2 incubator at 37 ºC. Twenty-four hours after seeding, scaffolds were collected for analysis. Harvested scaffolds were split into 5 equal sections approximately 6 mm long for analysis of seeding efficiency across the full length (Fig 2D). Section 1 represents the upstream entry, whereas section 5 is the distal end of the scaffold.

Seeding took place in three distinct methods: 1) bioreactor-mediated perfusion seeding, 2) static injection counterpart seeding, and 3) drip seeding.

#### 2.3.1 Bioreactor-mediated perfusion (BMP) seeding

Four scaffolds at the time were mounted into the custom-designed seed-and-culture bioreactor (Fig 2A). 10 mL of cell suspension, containing 2 x 10^6^ VSMCs, was drawn into sterile syringes and connected to the flow loop upstream of each scaffold. The tubing at the downstream end of each scaffold was clamped shut and the cell suspension was driven through the porous scaffold (Fig 2D) at a rate of 10 ml/hr with a syringe pump (New Era Pump Systems; Farmingdale, NY, USA).

#### 2.3.2 Static injection counterpart (SIC) seeding

Scaffolds were mounted onto modified stainless steel cannulas and filled with 100 μl of concentrated cell suspension containing approximately 2 x 10^6^ VSMCs (Fig 2B). Each end of the construct was closed and the scaffold submerged in full growth medium. This method replicated all the conditions of the perfusion-driven seeding method except the transmural pressure gradient supplied by the syringe pump.

#### 2.3.3 Drip seeding

Using standard aseptic procedures, 80 μl of a concentrated cell suspension containing approximately 2 x 10^6^ VSMCs was carefully pipetted directly onto the luminal surface of the tubular scaffolds (Fig 2C). Seeded scaffolds were placed in individual 100 mm culture dishes and incubated for 1 hour at 37°C. During this period the scaffold was rotated every 15 min to encourage even cell distribution. After one hour, scaffolds were submerged in full growth medium. This method replicated the target cell density of the previous methods, while depositing cells inside the scaffold manually.

### 2.4 Viability assessment and cell number

Scaffold sections produced for viability testing and seeding efficiency analysis were placed in 1000 μl of full growth medium supplemented with 100 μl of alamarBlue (BioRad; Hercules, CA, USA). The samples were incubated for 4 hours at 37 ºC with intermittent shaking. After incubation, 200 μl of the solution from each sample was placed into separate wells of a 96 well plate. Viable cells reduce resazurin, the active ingredient in alamarBlue, to resorfin which is highly fluorescent. This reaction was quantified with the hybrid microplate reader BioTek Synergy H1 (BioTek; Winooski, VT, USA). The cell number was obtained with a standard curve calculated using known concentration suspensions of cells from 25,000–275,000 cells in increments of 25,000.

Additionally, the spatial organization of viable cells was determined via a live/dead viability assay kit (BioVision, Waltham, MA, USA), which marks live and dead cells based on membrane integrity and esterase activity. Ethidium homodimer-1, enters cells with a compromised plasma membrane to bind DNA and emit a red fluorescence. Living cells are identified by Calcein AM, a fluorogenic dye that can permeate through the cell membrane to be converted to a green fluorescence after interaction with intracellular esterase. The middle section of 4 BMP -seeded scaffolds where harvested and incubated in a 1000:1:2 mixture of PBS, ethidium homodimer-1, Calcein AM for 15 minutes. ETVG circular cross-sections sections were cut into 3 sub-sections and grid-imaged from the luminal surface on a Nikon C2 confocal microscope system in z stacks of eight to twelve 16 μm slices. Images where stitched together using a 3D image stitcher plugin available through FIJI image manipulation software [16].

### 2.5 Immunohistochemical assessment

Scaffold sub-sections produced for spatial distribution analysis with haematoxylin and eosin stain (H&E) were fixed in 10% neutral buffered formalin overnight. Sections were stained *en bloc* with H&E and embedded in OCT Compound (Electron Microscopy Sciences; Hatfield, PA, USA) to cut 8 μm-thick cross-sections. Stained cross sections were imaged with an Eclipse LV100D microscope (Nikon Instruments; Melville, NY, USA).

Additional spatial distribution analysis was conducted by staining cell nuclei with DAPI. Scaffold sub-sections were harvested from the bioreactor and briefly rinsed in PBS before fixation in ice cold methanol for 5 minutes. Similar to the H&E samples, sections were stained *en bloc* before embedding in OCT medium and cutting. After fixation, scaffolds were rinsed in three 5-minute PBS baths. Membrane permeabilization was achieved by submersion in room temperature 0.1% Triton solution for 15 minutes. This was followed by another set of three PBS rinses and incubation with 300 nM DAPI solution for 5 minutes. Sections where imaged at 10x on the confocal microscope.

### 2.6 Immunohistochemical analysis

Raw H&E images were converted to binary with ImageJ software [17]. Binary images were post-processed to remove artifacts not relevant to analysis. Total cell load was determined by the total area of cells present (i.e. area of black pixels in the image). Circumferential cell coverage was calculated by measuring the length of the perimeter covered by cells and dividing by the total luminal perimeter of the scaffold. Average cell-layer thickness was determined by dividing the total cell load by the length of the perimeter covered by cells.

### 2.7 Scanning electron microscopy (SEM) assessment

Scaffold sections produced for surface coverage analysis were cut along the top most edge and placed in 10% neutral buffered formalin solution overnight. Sections were then rinsed 3x in PBS before dehydration in a series of graded ethanol (ETOH) washes from 50% to 100%. Chemical drying was achieved with graded washes of ETOH and hexamethyldisilane (HMDS) (MilliporeSigma; Burlington, MA, USA), followed by submersion in HMDS until fully evaporated.

After dehydration was completed, samples were mounted for imaging with the luminal side of the scaffold facing up and the circumferential direction corresponding to the horizontal direction of the sample. Samples were handled such that the middle section represents the bottom most section of the scaffold during seeding. Samples were gold sputter coated (Cressington 108, Ted Pella; Redding, CA, USA) and imaged with scanning electron microscopy (Phenom ProX Desktop SEM, NanoScience Instruments; Phoenix, AZ, USA) operated at 15kV. Imaging was conducted at 300x in a 3x3 grid across the full surface of the scaffold section to best approximate total surface coverage.

### 2.8 SEM analysis

Raw SEM images were converted to binary with the ImageJ plugin DiameterJ Segment [17]. Of the multiple binary images created from each raw SEM image, the one that was the most accurate in determining where cells were located (i.e. the black spaces in the image covered wherever cells were present in the original) was chosen for further evaluation. Another ImageJ function, Analyze Particles, was then employed for the quantification of percent area coverage.

Binarized images were checked to determine the general accuracy of the segmentation method before reporting. Accuracy was determined by randomly selecting 5 images from each seeding method and comparing results with cell coverage determined by manual segmentation.

### 2.9 Statistics

For all the analysis, three to four samples were used (n = 3+1). Values were reported as the average of all the samples, and the error was reported as either the standard deviation or 95% confidence interval (CI) in cases where the standard deviation was larger than the mean. The effect of seeding method and scaffold section within the seeding method on cell viability, surface coverage, and cell layer thickness were assessed with a standard ANOVA, and multiple pair-wise comparisons were carried out using the Tukey-HSD method. Differences were considered significant if p ≤ 0.05.

## 3. Results

### 3.1 Viability assessment and cell number

The seeding method had a significant effect on the number of viable cells present per section on electrospun PCL tubular scaffolds (p = 0.005). The average number of viable cells present per sample by method is 282,598 ± 53,957 cells, 237,220 ± 43,243 cells, and 163,929± 110,537 cells for BMP seeding, SIC seeding, and drip seeding, respectively. While these averages are not significantly different, the number of viable cells by section showed that perfusion-driven seeding results in significantly more cells by section, 56,519± 15,463 cells, than drip seeding, 42,182± 29,725 cells (p = 0.04). The average number of viable cells by section of SIC seeding, 47,444± 15,437 cells, was not significantly different than BMP seeding or drip seeding and the level of variability in longitudinal cell distribution between samples was high.

**Fig 3:**
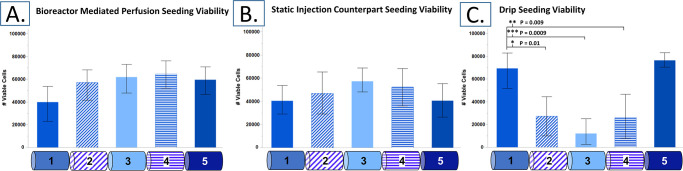
AlamarBlue viability assay results. Section average viable cell numbers for (A) perfusion bioreactor seeding, (B) static counterpart seeding, and (C) drip seeding. (n = 4; mean with 95% confidence intervals).

Perfusion-driven seeding also resulted in the most consistent pattern of cell distribution of the three methods. While section 1 consistently resulted in lower numbers of viable cells than sections 2–5, sections 2–5 were indistinguishable (with p > 0.95, Fig 3A). SIC seeding also showed no significant difference between sections and when averaged showed a normal distribution pattern with higher concentration of cells in section 3, the middle of the scaffold (Fig 3B). The standard deviation between SIC samples was larger than in BMP seeded samples, showing that while there is no observable significant difference between averages, the results were not as consistent. Drip seeded scaffolds did have a distinct pattern of cell distribution identifiable in all samples analyzed (Fig 3C). Drip seeding did not achieve uniformity across the entire length of the scaffolds, the majority of cells were found at either end of the scaffolds, sections 1 and 5, with very few to be found in the middle section. Sections 1 and 5 were found to be significantly different from sections 2, 3 and 4 (p = 0.01, 0.0009, 0.009 and p = 0.03, 0.0003, 0.003 respectively, the latter set not included in Fig 3C).

**Fig 4:**
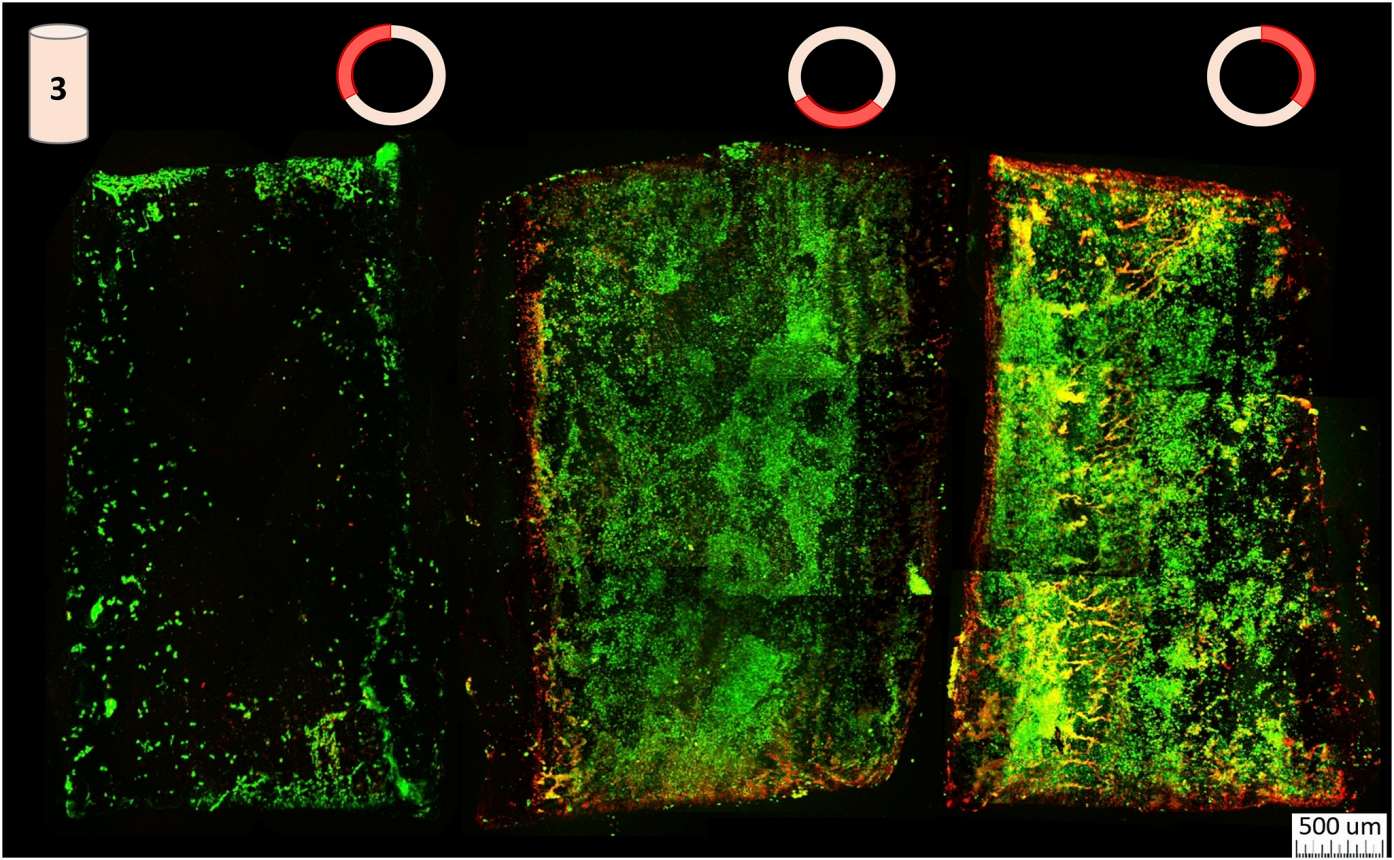
Spatial live/dead cell imaging. The middle section of a perfusion seeded scaffold is represented in 3rds. The left most portion is the top right edge of the scaffold, the middle portion is the bottom of the scaffold, and the right most portion is the top left side of the scaffold. Each section length was approximately 5 mm, and its circumference was 6.28 mm.

The live dead cell imaging of bioreactor mediated perfusion seeded scaffolds revealed a thick, confluent layer of living cells covering approximately two thirds of the scaffold luminal surface (Fig 4). The 4 other scaffolds evaluated this way also follow this pattern (S1 Fig). Non-viable cells can be found amongst the live cells, but they are mostly concentrated near the edges where the scaffolds sub-sections were cut, and along major defects in the scaffold structure. The third section of the scaffold that does not contain a confluent layer of cells shows small live cell clumps interspersed throughout the area.

In summary, BMP seeding resulted in the most viable cells with the most consistent distribution across the samples. SIC seeding resulted in a comparable number of cells but with a more inconsistent distribution, and drip seeding resulted in the least number of cells but a high degree of consistency in distribution pattern that featured most of the cells focalized at either end, and middle sections remaining bare.

### 3.2 Immunohistochemical spatial distribution analysis

**Fig 5:**
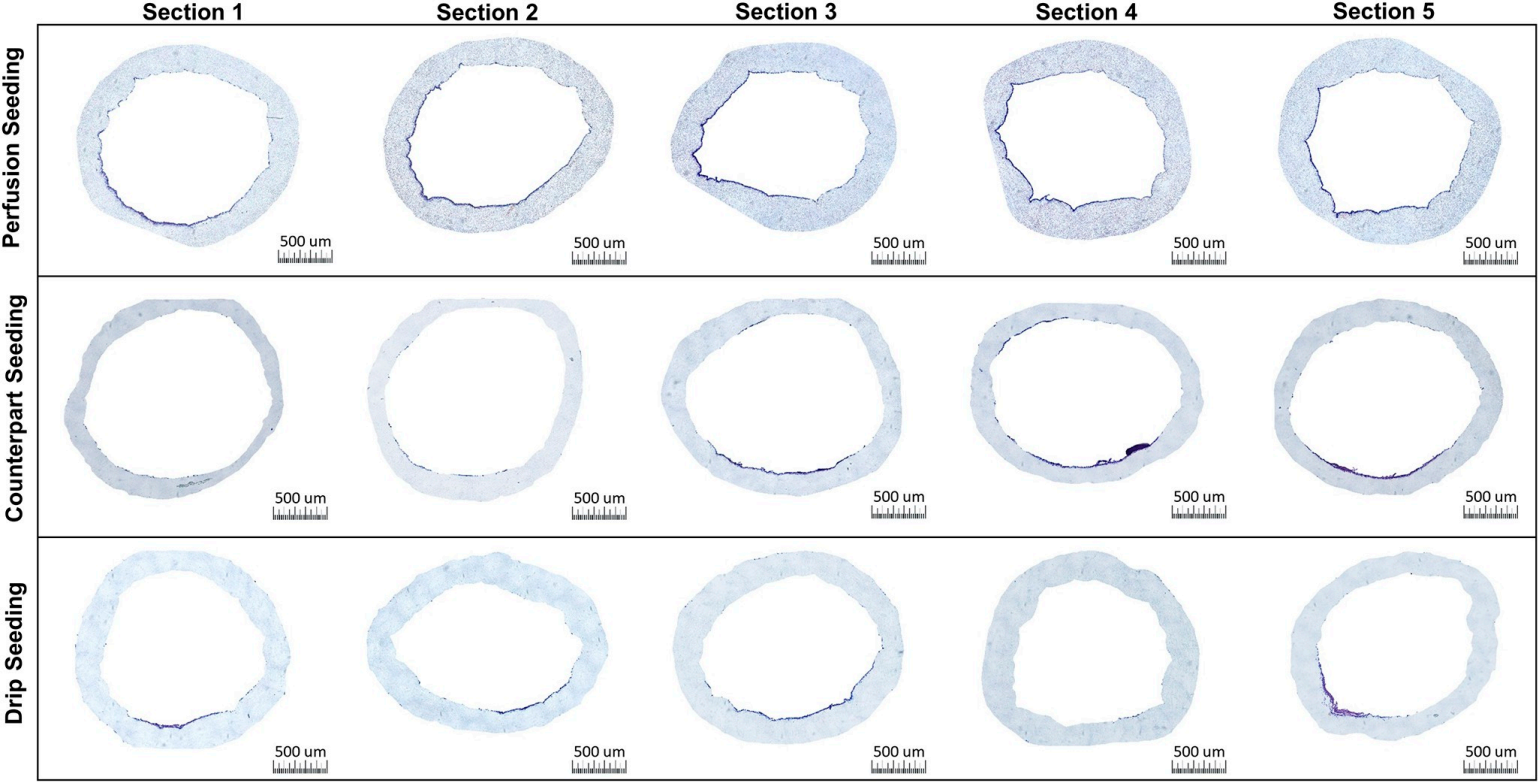
H&E cross-sections. Representative haematoxylin and eosin (H&E) stained cross sections of each seeding method 24 hrs after seeding in longitudinal order.

BMP seeding resulted in the most consistent cell coverage across the five sections in terms of cell presence around the circumference, though cell-layer thickness varied spatially (Fig 5). Both the SIC and drip seeded samples resulted in cells restricted to a few smaller locations, with inconsistent coverage between sections, and variable thickness of cell layers within each section, often settling in clumps and large regions remaining bare (S2 Fig). Drip seeding in particular resulted in multiple sections entirely without cells, usually sections 2–4. Due to such, quantitative analysis was conducted on the BMP and SIC seeded scaffolds only. Perfusion-driven seeding consistently showed cells dispersed across about 60% of the scaffold area, with the highest concentration being located along the bottom edge of the scaffold. BMP seeding also resulted in sporadic cell patches occasionally adhered to the top edge of the scaffold, something rarely seen in SIC or drip seeding methods.

**Fig 6:**
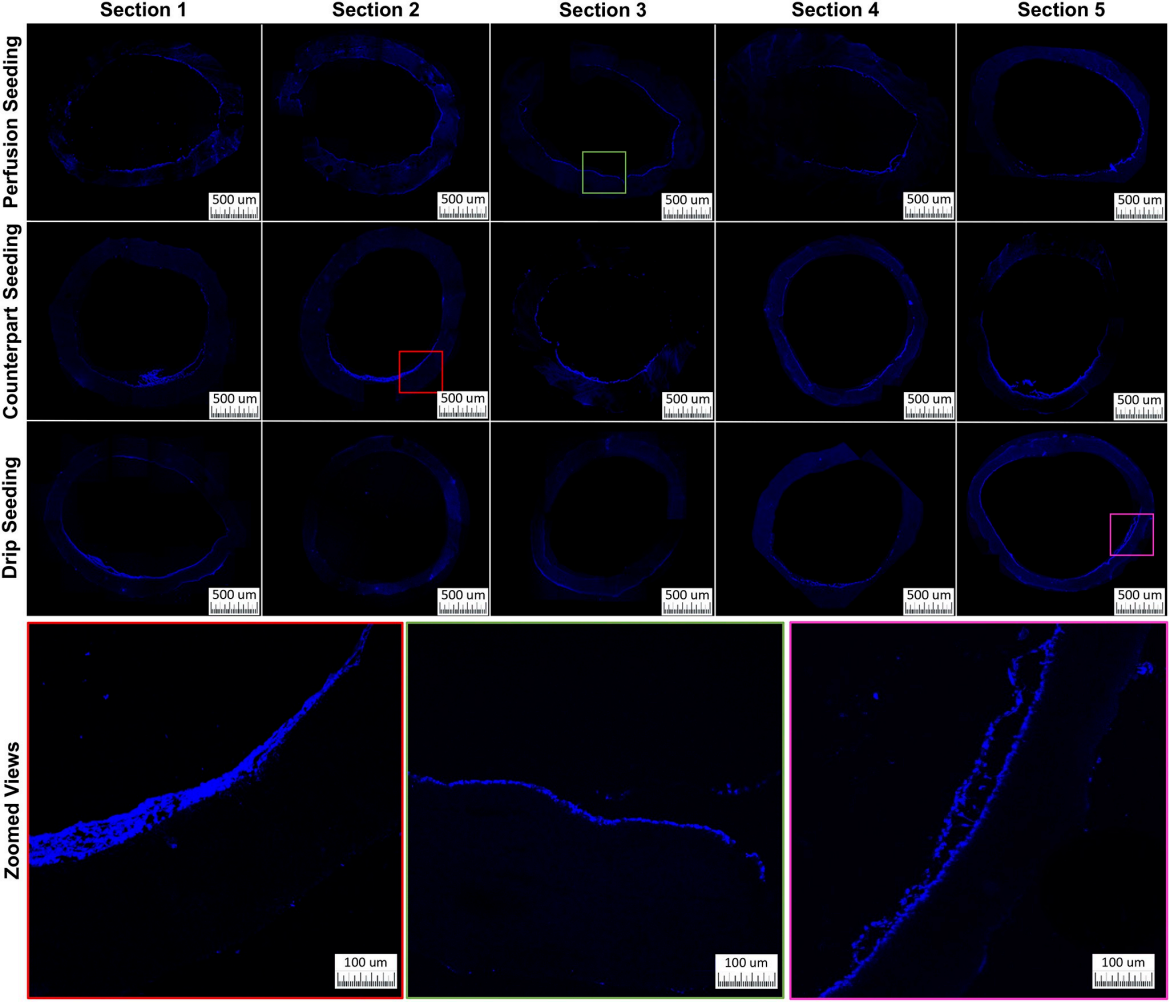
DAPI cross-sections. DAPI stained nuclei of cells in cross sections of each seeding method 24 hrs after seeding in longitudinal order.

DAPI stained cross sections show a similar distribution of cells when compared to H&E stained sections, with emphasis on individual nuclei (Fig 6). This confirms that the cell layer deposited by bioreactor-mediated perfusion-seeding is indeed composed by cells with DAPI stainable nuclei and typically one layer thick covering > 60% of the luminal surface. DAPI results obtained with both static methods corroborate H&E histology showing inconsistency in the cell layer thickness and circumferential distribution between sections.

**Fig 7:**
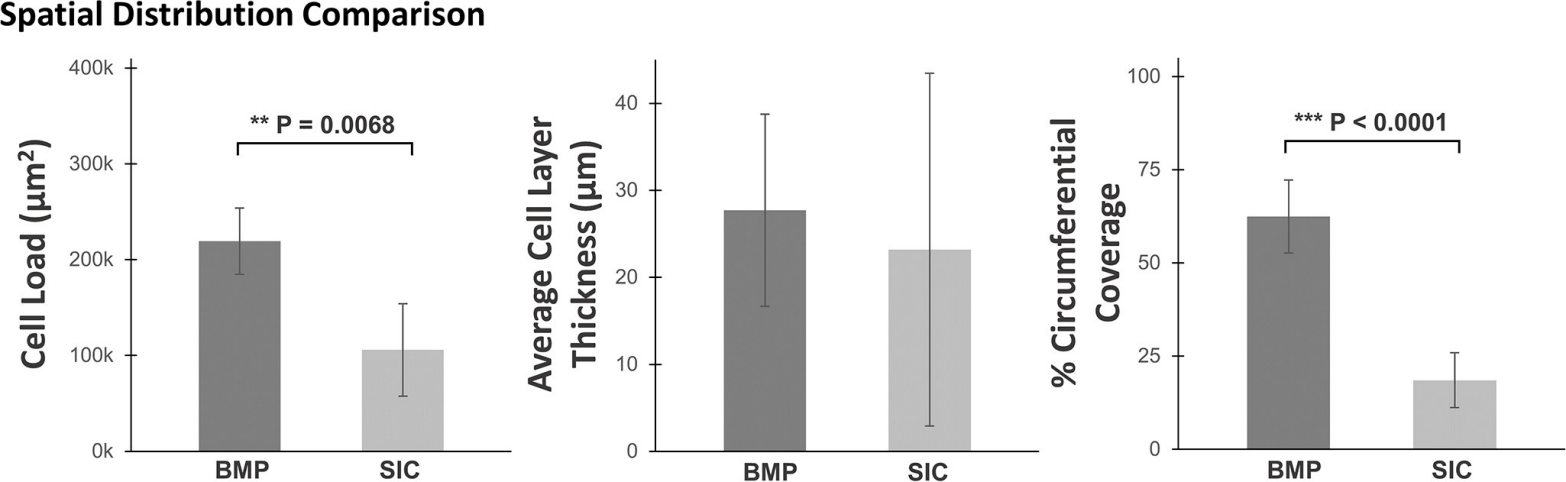
Quantitative assessment of uniformity. Quantitative analysis of H&E images for bioreactor mediated perfusion and static injection counterpart seeding methods preformed with rVSMCs onto electrospun PCL scaffolds. Uniformity judged in terms of total cell load, average cell layer thickness, and % circumferential coverage per section. (n = 20, n = 15; mean ± standard deviation).

Quantitative analysis of histology sections largely agrees with viability results. BMP seeding resulted in a significantly higher total cell load being deposited to the scaffold lumen than SIC seeding (p = 0.007, Fig 7). The variability of total cell load and average cell-layer thickness between sections in the SIC seeded samples corroborates with variations observed in the cell viability analysis (Fig 3B). Though there was no significant difference in the average cell layer thickness between the two methods (27.8 vs 29.1 μm), the standard deviation was observed to be larger for individual sections in the SIC method. The proportion (or percentage) of circumferential coverage between BMP seeding and SIC seeding was significantly different (p<0.0001), on average achieving 62.8± 20.7% and 23.6± 15.2% respectively. In summary, BMP seeding achieves significantly more cell load and percent circumferential coverage than static methods while also maintaining a consistent cell layer thickness and distribution between all five sections.

### 3.3 SEM surface coverage analysis

**Fig 8:**
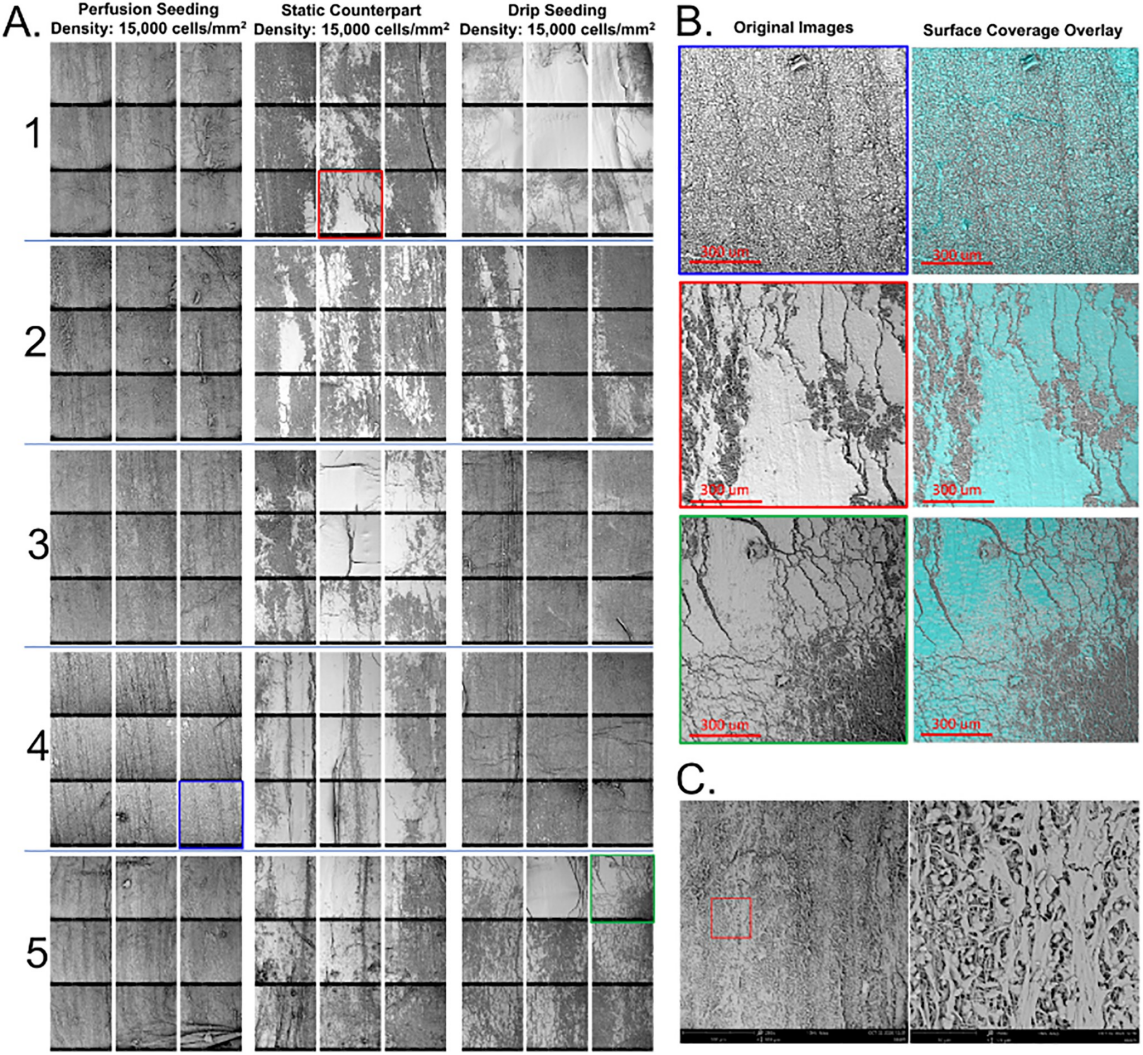
Interpretation of SEM images. (A) Representative SEM images of luminal surface of seeded electrospun PCL scaffolds for each seeding method, (B) Sample images representing quantitative analysis of luminal surface coverage per method. (C) Zoomed in view of perfusion seeded scaffold.

Analysis of the topography of scaffold sections agrees with viability and histology results. BMP seeding showed consistent coverage across the full scaffold surface (Fig 8A) Additionally, perfusion-driven seeding resulted in individual cells spaced apart from each other while both static methods led to clumps of cell aggregates from which individual cells could not be identified in the SEM images (Fig 8B). Cells tend to concentrate along the bottom of the scaffold (middle column of the SEM 3x3 sample views) in the SIC method, and drip seeded scaffolds showed large concentrations of cells at the ends while typically achieving fuller surface coverage in these sections. BMP seeded cells did not appear to be damaged from the perfusion treatment, the morphology of the individual cells qualitatively showed evidence of adherence to the scaffold surface (Fig 8C).

**Fig 9:**
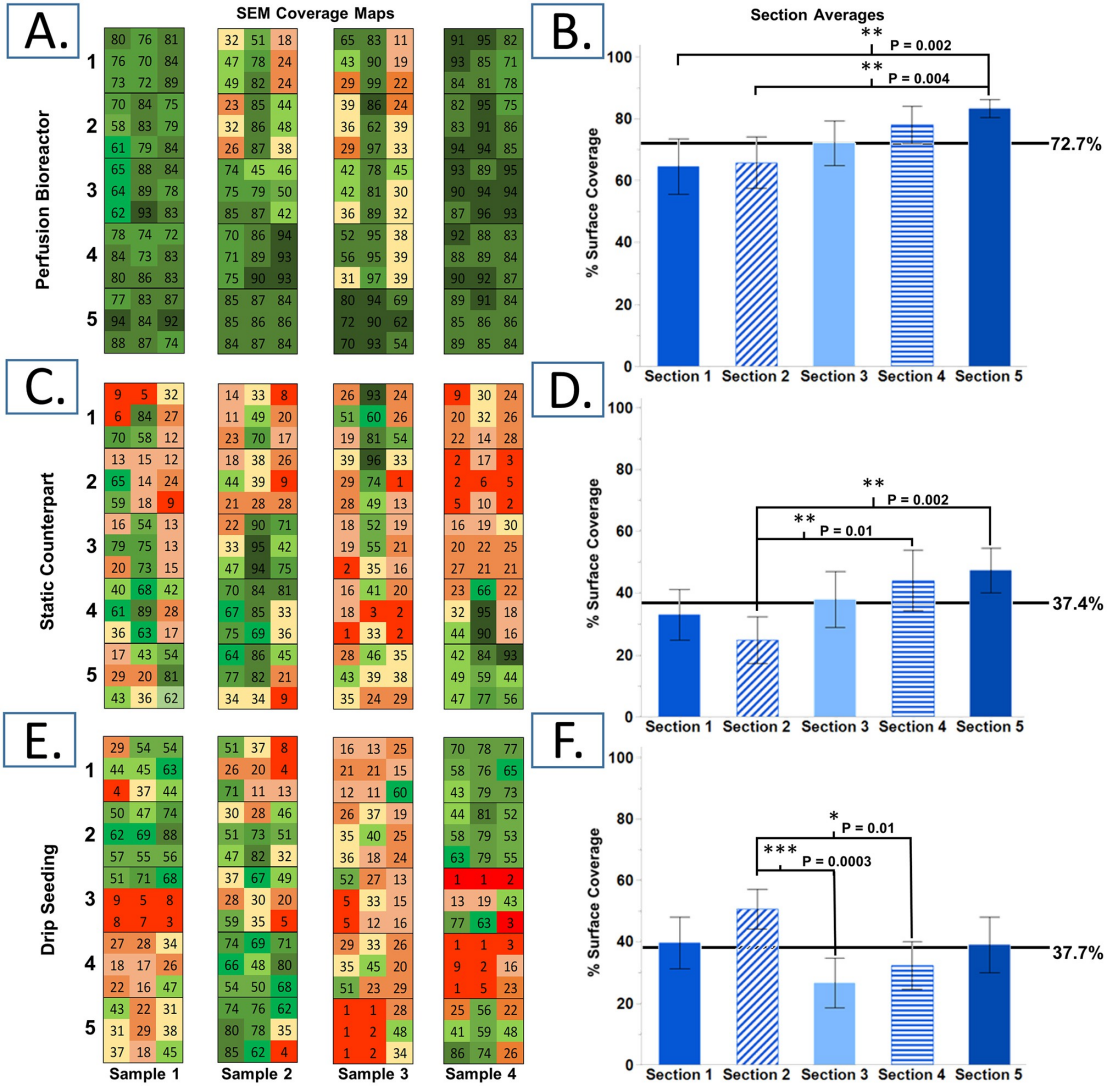
Quantitative analysis of surface coverage. Color map of surface coverage and section averages respectively for (A,B) perfusion bioreactor seeding, (C,D) static counterpart seeding, and (E,F) drip seeding (n = 36; mean with 95% confidence intervals).

Quantitative analysis shows that BMP seeding resulted in significantly higher surface coverage across the scaffold surface, 72.7± 21.7%, when compared to static methods, which averaged 37.4± 25.8% and 37.7± 24.7% (p<0.0001, Fig 9A vs. 9C and 9E respectively). BMP seeded sections did reveal a gradient of increasing surface coverage from sections 1 to 5, i.e. upstream to downstream. Sections 1 and 2 on average achieved significantly less surface coverage than section 5 (p = 0.002 and p = 0.004 respectively, Fig 9B). Though more inconsistent than previous results suggest, the average surface coverage of sections 1 and 2 are 64.6± 26.32% and 65.8± 24.5% respectively, which is still higher than the approximately 37% surface coverage achieved by either static method per section on average. The percent area coverage in SIC and drip seeded scaffolds were variable between sample sections. The distribution pattern for SIC scaffolds tends to be along the bottom edge of the scaffold with patches of high cell density, while drip seeded scaffolds tended to have more surface coverage at either end. Both static methods show a high level of intra-sample variability in cell distribution, either along the length or the circumference of the scaffolds (Fig 9C–9F). Analysis of accuracy showed that manual segmentation vs. automatic segmentation did not deviate more than 2% in the 15 random SEM images analyzed.

### 3.4 Combined results

**Fig 10:**
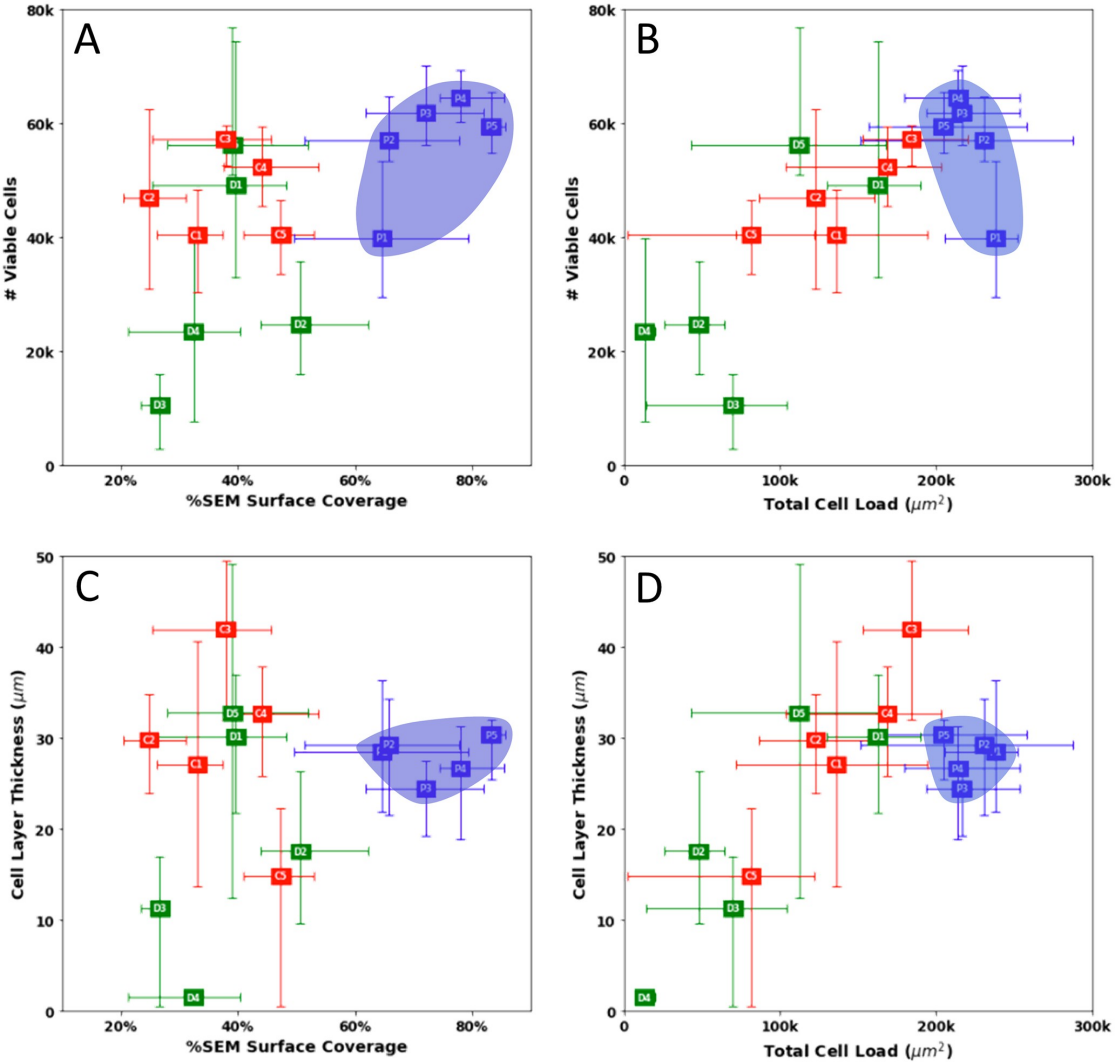
Scatter plots of combined data. The top left graph compares the average number of viable cells present on each scaffold section to the surface coverage determined with SEM analysis. The top right graph compares the number of viable cells and the cell load area as determined by H&E analysis. The bottom left graph compares the average cell layer thickness to SEM surface coverage and the bottom right graph compares cell layer thickness to cell load. Bioreactor mediated perfusion data is highlighted in blue.

Establishing correlations between the data reveals that BMP seeding resulted in the most consistent seeding pattern across all five sections (grouped in blue in Fig 10). When comparing the number of viable cells to percent surface coverage, perfusion-driven seeded scaffolds outperformed static methods in both aspects, achieving comparable viable cell load with higher cell coverage (> 60% vs. < 50% of either static method, Fig 10A). Comparing the number of viable cells obtained from the alamarBlue viability assay to the total cell load determined by histology resulted in a linear correlation (Fig 10B). Correlation of histology determined cell load or SEM-determined surface coverage vs. cell-layer thickness demonstrated again the improved performance and consistency achieved with perfusion-driven seeding method compared to static methods (Fig 10C and 10D).

## 4. Discussion

While clinical application of ETVGs has been limited, years of testing has made it clear what characteristics an ETVG needs to maintain patency [18]. Successful vascular grafts require a non-thrombogenic luminal surface, robust mechanical properties, and the ability to remodel in vivo, and each of these requirements is affected by the presence of cells both during development and after implantation [19–21]. It is imperative that the seeding method used for such constructs be simple and efficient so the resulting engineered tissue can develop quickly and homogenously on the scaffold.

Seeding of tubular scaffolds has been typically achieved by pipetting the endothelial cells directly onto the scaffold surface, followed by culture in bioreactor systems that mimic hemodynamic pressure aspects and prepare ETVGs for in vivo function when exposed to blood, reducing its potential thrombogenicity and improving patency. However, this technique is limited by its poor efficiency and possible cell detachment when exposed to flow. Some studies utilizing drip seeding reported cell loss of 70% after a few minutes and up to 95% in the first 24 hours after implantation [22]. Many different techniques to improve seeding efficiency and patency outcomes have been developed since then such as special formulations of culture medium, single-stage vs. multi-stage seeding, and the inclusion of mechanical/hemodynamic stresses [23]. Extensive empirical experimentation in the field of vascular tissue engineering has discovered no clear potential benefits from one seeding method over others. Many of the studies have different goals associated with their experimentation, which has great influence on the choice of seeding method employed. This study attempts a bona-fide comparison in between three different seeding methods, each taking similar amounts of time and effort to complete. No additives were used to enhance or facilitate cell adhesion to the scaffolds. Each of these methods can be accomplished for extremely low cost, especially when considering that the syringe pump included in the BMP seeding method could feasibly be substituted with a syringe featuring a high friction coefficient and the gravitational force of a properly applied weight. Judging the three seeding methods to be equitable in cost, complexity, and time consumption, the question remains if any has a clear benefit over the others.

Automatic seeding within bioreactors has the benefit of limiting the manual handling of scaffolds, which in turn limits possible contamination scenarios. Due to this major benefit, it has been investigated repeatedly since its initial development in 1990 by Wildevuur et al. [24]. Originally, this method was developed as a quick way to seed vascular grafts during the operating procedure at the patient’s bedside. In one exploratory study by Noishiki et al., an 8 cm section of vasculature was finely minced and suspended in 20 ml of physiological saline solution. The resulting suspension was pushed transmurally through the lumen of a highly porous fabric scaffold via repeated pressurized injection [25]. After implantation in a canine model, it was reported that the grafts experienced increased thrombus formation; however, localization of cell types to their natural physiological locations was also observed and after 14 days the grafts were reported to be fully endothelialized. This result was initially promising, but the study failed to address the rapid transanastomotic endothelial outgrowth observed in animals, and the requirement of a sacrificial vessel fragment for the seeding does not reduce patient risk associated with multiple surgeries.

Since then, several groups have developed and employed other perfusion-based methods to create small diameter ETVGs. Feijen and co-workers have cannulated porous tubular polytrimethylene carbonate scaffolds with 3mm inner diameter in custom-made glass flow chambers and injected 20 ml of SMC cell suspension through their lumen [26, 27]. The cell suspension was injected with two syringes, one positioned at either end of the scaffold. Scaffolds were manually rotated every 30–60 min during the first 2.5 hours after seeding to promote homogeneous cell adhesion, and cultured with either static of dynamic flow conditions up to 14 days. Cell presence after 7 days was observed transmurally throughout the bulk of the scaffold. However, a full analysis along the scaffold length or circumference was not demonstrated, so more detailed spatial distributions of cell seeding could not be determined. Furthermore, rotating after injection when dealing with thick, porous scaffolds, is counter intuitive, as a dense pore network may impede cell migration due to gravity. This is supported by the study observation that thicker scaffolds took a longer time to seed, suggesting the structure impeded cell movement within the scaffold thickness. If in fact the study achieved a homogenous circumferential distribution, it would be interesting to see if a similar result could be achieved without the rotation period.

Vorp and colleagues have developed and refined methods for seeding of vascular grafts that combine double injection filtration perfusion, vacuum perfusion and a rotational element concurrently [4, 28]. Studies were conducted to examined the effectiveness of the seeding method on two distinct scaffold types [28]. One had small pore sizes and infiltration into the scaffold thickness was limited, while the other contained larger pores and allowed cellular infiltration. Cell distribution across the full length of the scaffold was investigated and found to be homogenous and viable in both cases. Furthermore in 2020, Cunnane et al. revised the system to accommodate grafts with lengths comparable to those used for human arterial replacement [29]. Instead of two syringes seeding the graft at both ends, a diffuser was inserted into the scaffold lumen. Over the course of the seeding period, the diffuser would translate across the graft length while dispensing cell suspension from the distal end. Results indicated that this method also achieved a uniform distribution both longitudinally and circumferentially with a scaffold six times the length of those in the previous study (12cm vs 2cm). While the results are overwhelmingly positive, the complexity of the seeding systems and experimental protocol limits further manipulation of the scaffold environment after seeding as well as limiting similar application to systems that attempt to culture more than one graft at a time.

To our knowledge, the bioreactor system presented here is the first to be developed that incorporates semi-automated cell seeding and subsequent culture in a system conducive to subject biaxial mechanical stimulation on four small diameter tubular scaffolds simultaneously. The ability to conduct tissue engineering experiments in a cost- and resource-effective experimental program is crucial to refine methods and obtain reproducible and statistically reliable data to provide a better mechanistic understanding of engineered tissue growth and remodeling during its in vitro incubation stage.

Seeded scaffolds were evaluated longitudinally and circumferentially in terms of cell load, viability, cell layer thickness, and surface coverage distribution. BMP seeding resulted in comparable numbers of viable cells as static drip seeding and the SIC methods. However, the distribution of the cells present varied greatly between methods. Drip seeding onto the luminal surface resulted in high concentrations of cells at either end of the scaffold and very few in the middle, as expected. The SIC and BMP seeding had similar longitudinal cell distributions, but static injection resulted in 35% less circumferential coverage on average. Interestingly, our histology analysis revealed a more significant difference between BMP and static seeding methods in terms of cell load than suggested by the results obtained in our alamarBlue viability assay. We surmise that the histology washes may have washed away some of the cells present after static seeding. BMP seeding was seemingly unaffected by this possible phenomenon, leading us to believe that perfusion not only leads to better cellular coverage but also enhances cell attachment to our scaffolds.

Earlier attempts at creating ETVGs have used seeding densities anywhere between 2,000 and 45,000 cells/mm^2^ [24, 30]; however since the early 2000s, cell densities between 10,000 and 20,000 cells/mm^2^ [27, 31–33] are typically employed. In this experiment, 2 x 10^6^ cells were seeded onto each scaffold, a target seeding density of 10,610 cell/mm^2^, after seeding the viable cell density was calculated to be about 1,600 cell/mm^2^ which is comparable to the results previously achieved and published in the literature by others [24, 27, 33]. Scaffolds seeded with our bioreactor system demonstrated that cell coverage was lesser in the first 6 mm of length proximal to the solution input. We believe this may be due to the pressurization pattern during seeding and could be mitigated by the addition of a second syringe perfusing through the distal end of the scaffold. However, the benefit of such an addition could be negligible. The differences between methods warrants further investigation in terms of how this effects long term culture patterns.

Differences in apparent SMC morphology perceived from close inspection of the SEM images could be related with the phenotypic modulation of SMCs, which could change from synthetic to contractile if part of a large aggregate as seen in the denser regions of drip seeded and statically infused scaffolds. Contractile SMCs are elongated, spindle-shaped cells, whereas synthetic/proliferative SMCs are less elongated and have a cobblestone morphology [34]. However, SEM imaging is purely topographical and the boundary between cells can be difficult to define if cells form aggregates with multiple layers and have deposited ECM into the interstitial space between cells. In the bioreactor mediated perfusion seeding method, cells are distributed more homogenously and typically in a monolayer, with different degrees of sparsity, so it is easier to distinguish individual cells from one another. In the other static methods, cells would typically aggregate in a small region of the scaffold area which presents itself as a smooth surface with individual cells difficult to identify.

There are many limitations associated with the work presented here as there often are with ETVG-related experiments. To determine feasibility in the clinical setting, the system would need to be scaled up to the appropriate size. Vascular grafts for human CABG are typically 5 mm in diameter and 18 cm long resulting in an aspect ratio of 45, compared to the aspect ratio employed here, which is about 20 [35, 36]. It is well known that ETVGs with substantial synthetic polymer component typically do not perform well in long term in vivo studies due to issues associated with compliance mismatch [37]. However, the BMP seeding method presented here could easily fit scaffolds of different materials, microstructures and sizes with little to no modification. The scaffolds used in this study were developed in house with reasonable consistency and throughput, and eliminated dependence on outside suppliers. Certainly, this study would benefit with the analysis of different scaffold types with different microstructures to verify if the benefits of bioreactor-mediated perfusion seeding would be conserved. However, this study was centered around the characterization of cell delivery onto scaffolds by the system. Additional determinations of cell morphology, such as staining with vascular SMC contractile markers like alpha smooth muscle actin (α-SMA), smoothelin, or smooth muscle myosin heavy chain (SM-MHC) would provide more information towards determining how the seeding process may affect SMC phenotype and behavior relevant for the subsequent steps of in vitro culture. Furthermore, we have looked into additive agents that are used to prevent cell sedimentation in syringes and tubing as well as various proteins that scaffolds may be coated with to promote cell adherence, but none were used in this study, which may be considered an oversite when determining the effectiveness of our method. Additive agents in 3D culture media can be expensive, and there are a great number to choose from. We believe that the positive results obtained in this study are encouraging and promote the possibility of future studies to tailor those aspects to address a variety of problems.

## 5. Conclusion

In this work, we presented the validation of the seeding method associated with a custom designed seed and culture bioreactor for the development of ETVGs under the influence of cyclic biaxial mechanical stimulation. Our method seeds cells onto the luminal surface of electrospun polymer scaffolds in a demonstrably consistent manner when compared to traditional static seeding methods. It also eliminates the need for excessive handling during the transition in between seeding and culturing. This device may contribute to the progress of any small diameter tubular tissue engineering application that aims to investigate the effect of mechanical stimulation on tissue development.

## Supporting information

S1 Fig Live/dead cell imaging complete results. The middle section of the 3 additional perfusion seeded scaffolds are represented in 3rds. Living cells are stained green while dead cells are stained red. View Fig 4 for orientation.
(TIFF)

S2 Fig Raw histology images and ImageJ binary edits. (TIFF)

S3 Fig Bioreactor mediated perfusion raw SEM images. (TIFF)

S4 Fig Static injection counterpart raw SEM images. (TIFF)

S5 Fig Drip raw SEM images. (TIFF)

S6 Fig Histology data. Measured histology data values of individual scaffold sections for (A) bioreactor-mediated perfusion seeded grafts and (B) static-injection counterpart seeded grafts.
(TIFF)

S7 Fig Viability data graphs for individual samples. Measured viability data values of individual scaffold sections for (A) bioreactor-mediated perfusion seeded grafts, (B) static-injection counterpart seeded grafts and (C) drip seeded grafts.
(TIFF)

S1 Table Histology analysis section averages and standard deviations including drip seeded scaffolds. (PDF)

S2 Table SEM analysis error evaluation. (PDF)

## References

[1] Mensah GA, Brown DW. An overview of cardiovascular disease burden in the United States. Health Aff. 2007;26(1):38–48. doi: 10.1377/hlthaff.26.1.38 17211012

[2] Conklin BS, Richter ER, Kreutziger KL, Zhong DS, Chen C. Development and evaluation of a novel decellularized vascular xenograft. Med Eng Phys. 2002;24(3):173–83. doi: 10.1016/s1350-4533(02)00010-3 12062176

[3] Dean EW, Udelsman B, Breuer CK. Current Advances in the Translation of Vascular Tissue Engineering to the Treatment of Pediatric Congenital Heart Disease. Yale J Biol Med. 2012;85(2):229–38. 22737051 PMC3375656

[4] Soletti L, Nieponice A, Guan J, Stankus JJ, Wagner WR, Vorp DA. A seeding device for tissue engineered tubular structures. Biomaterials. 2006;27(28):4863–70. doi: 10.1016/j.biomaterials.2006.04.042 16765436

[5] Tara S, Rocco KA, Hibino N, Sugiura T, Kurobe H, Breuer CK, et al. Vessel bioengineering—Development of small-diameter arterial grafts -. Circ J. 2014;78(1):12–9. doi: 10.1253/circj.cj-13-1440 24334558

[6] Atala A, Kurtis Kasper F, Mikos AG. Engineering complex tissues. Sci Transl Med. 2012;4(160):1–11. doi: 10.1126/scitranslmed.3004890 23152327

[7] Li Y, Ma T, Kniss DA, Lasky LC, Yang ST. Effects of filtration seeding on cell density, spatial distribution, and proliferation in nonwoven fibrous matrices. Biotechnol Prog. 2001;17(5):935–44. doi: 10.1021/bp0100878 11587587

[8] Villalona GA, Udelsman B, Duncan DR, McGillicuddy E, Sawh-Martinez RF, Hibino N, et al. Cell-seeding techniques in vascular tissue engineering. Tissue Eng—Part B Rev. 2010;16(3):341–50. doi: 10.1089/ten.TEB.2009.0527 20085439 PMC2946885

[9] Burg KJL, Holder WD, Culberson CR, Beiler RJ, Greene KG, Loebsack AB, et al. Comparative study of seeding methods for three-dimensional polymeric scaffolds. J Biomed Mater Res. 2000;51(4):642–9. doi: 10.1002/1097-4636(20000915)51:4&lt;642::aid-jbm12&gt;3.0.co;2-l 10880112

[10] Shimizu K, Ito A, Arinobe M, Murase Y, Iwata Y, Narita Y, et al. Effective cell-seeding technique using magnetite nanoparticles and magnetic force onto decellularized blood vessels for vascular tissue engineering. J Biosci Bioeng. 2007;103(5):472–8. doi: 10.1263/jbb.103.472 17609164

[11] Perea H, Aigner J, Hopfner U, Wintermantel E. Direct magnetic tubular cell seeding: A novel approach for vascular tissue engineering. Cells Tissues Organs. 2006;183(3):156–65. doi: 10.1159/000095989 17108686

[12] Martin I, Wendt D, Heberer M. The role of bioreactors in tissue engineering. Trends Biotechnol. 2004;22(2):80–6. doi: 10.1016/j.tibtech.2003.12.001 14757042

[13] Patel NM, Yazdi IK, Tasciotti E, Birla RK. Optimizing cell seeding and retention in a three-dimensional bioengineered cardiac ventricle: The two-stage cellularization model. Biotechnol Bioeng. 2016;113(10):2275–85. doi: 10.1002/bit.25992 27071026

[14] Yeatts AB, Fisher JP. Bone tissue engineering bioreactors: Dynamic culture and the influence of shear stress. Bone. 2011;48(2):171–81. doi: 10.1016/j.bone.2010.09.138 20932947

[15] Wendt D, Marsano A, Jakob M, Heberer M, Martin I. Oscillating perfusion of cell suspensions through three-dimensional scaffolds enhances cell seeding efficiency and uniformity. Biotechnol Bioeng. 2003;84(2):205–14. doi: 10.1002/bit.10759 12966577

[16] Preibisch S, Saalfeld S, Tomancak P. Globally optimal stitching of tiled 3D microscopic image acquisitions. Bioinformatics. 2009;25(11):1463–5. doi: 10.1093/bioinformatics/btp184 19346324 PMC2682522

[17] Hotaling NA, Bharti K, Kriel H, Simon CG. DiameterJ: A validated open source nanofiber diameter measurement tool. Biomaterials. 2015;61:327–38. doi: 10.1016/j.biomaterials.2015.05.015 26043061 PMC4492344

[18] Zilla P, Deutsch M, Bezuidenhout D, Davies NH, Pennel T. Progressive Reinvention or Destination Lost? Half a Century of Cardiovascular Tissue Engineering. Front Cardiovasc Med. 2020;7(September):1–32. doi: 10.3389/fcvm.2020.00159 33033720 PMC7509093

[19] Skovrind I, Harvald EB, Juul Belling H, Jørgensen CD, Lindholt JS, Andersen DC. Concise Review: Patency of Small-Diameter Tissue-Engineered Vascular Grafts: A Meta-Analysis of Preclinical Trials. Stem Cells Transl Med. 2019;8(7):671–80. doi: 10.1002/sctm.18-0287 30920771 PMC6591545

[20] Niklason LE, Lawson JH. Bioengineered human blood vessels. Science. 2020;370(6513). doi: 10.1126/science.aaw8682 33033191

[21] Gupta P, Mandal BB. Tissue-Engineered Vascular Grafts: Emerging Trends and Technologies. Adv Funct Mater. 2021;31(33):1–28. doi: 10.1002/adfm.202100027

[22] Sánchez PF, Brey EM, Briceño JC. Endothelialization mechanisms in vascular grafts. J Tissue Eng Regen Med. 2018;12(11):2164–78. doi: 10.1002/term.2747 30079631

[23] Zhang WJ, Liu W, Cui L, Cao Y. Tissue engineering of blood vessel: Tissue Engineering Review Series. J Cell Mol Med. 2007;11(5):945–57. doi: 10.1111/j.1582-4934.2007.00099.x 17979876 PMC4401266

[24] van Wachem PB, Stronck JWS, Koers-Zuideveld R, Dijk F, Wildevuur CRH. Vacuum cell seeding: a new method for the fast application of an evenly distributed cell layer on porous vascular grafts. Biomaterials. 1990;11(8):602–6. doi: 10.1016/0142-9612(90)90086-6 2279063

[25] Noishiki Y, Yamane Y, Tomizawa Y, Okoshi T, Satoh S, Wildevuur CRH, et al. Rapid endothelialization of vascular prostheses by seeding autologous venous tissue fragments. J Thorac Cardiovasc Surg. 1992;104(3):770–8. doi: 10.1016/s0022-5223(19)34749-x 1513165

[26] Engbers-Buijtenhuijs P, Buttafoco L, Poot AA, Dijkstra PJ, De Vos RAI, Sterk LMT, et al. Biological characterisation of vascular grafts cultured in a bioreactor. Biomaterials. 2006;27(11):2390–7. doi: 10.1016/j.biomaterials.2005.10.016 16343614

[27] Song Y, Wennink JWH, Kamphuis MMJ, Vermes I, Poot AA, Feijen J, et al. Effective seeding of smooth muscle cells into tubular poly(trimethylene carbonate) scaffolds for vascular tissue engineering. J Biomed Mater Res Part A. 2010;95A(2):440–6. doi: 10.1002/jbm.a.32859 20648539

[28] Nieponice A, Soletti L, Guan J, Deasy BM, Huard J, Wagner WR, et al. Development of a tissue engineered vascular graft combining a biodegradable scaffold, muscle-derived stem cells and a rotational vacuum seeding technique. Biomaterials. 2008;29(7):825–33. doi: 10.1016/j.biomaterials.2007.10.044 18035412 PMC2354918

[29] Cunnane EM, Lorentz KL, Soletti L, Ramaswamy AK, Chung TK, Haskett DG, et al. Development of a Semi-Automated, Bulk Seeding Device for Large Animal Model Implantation of Tissue Engineered Vascular Grafts. Front Bioeng Biotechnol. 2020;8(October):1–10. doi: 10.3389/fbioe.2020.597847 33195168 PMC7644804

[30] Hoerstrup SP, Zünd G, Sodian R, Schnell AM, Grünenfelder J, Turina MI. Tissue engineering of small caliber vascular grafts. Eur J Cardio-thoracic Surg. 2001;20(1):164–9. doi: 10.1016/s1010-7940(01)00706-0 11423291

[31] Vaz CM, van Tuijl S, Bouten CVC, Baaijens FPT. Design of scaffolds for blood vessel tissue engineering using a multi-layering electrospinning technique. Acta Biomater. 2005;1(5):575–82. doi: 10.1016/j.actbio.2005.06.006 16701837

[32] Roh JD, Nelson GN, Brennan MP, Mirensky TL, Yi T, Hazlett TF, et al. Small-diameter biodegradable scaffolds for functional vascular tissue engineering in the mouse model. Biomaterials. 2008;29(10):1454–63. doi: 10.1016/j.biomaterials.2007.11.041 18164056 PMC2375856

[33] Huang AH, Niklason LE. Engineering biological-based vascular grafts using a pulsatile bioreactor. J Vis Exp. 2011;1(52):5–10. doi: 10.3791/2646 21694696 PMC3197033

[34] Rensen SSM, Doevendans PAFM, Van Eys GJJM. Regulation and characteristics of vascular smooth muscle cell phenotypic diversity. Netherlands Hear J. 2007;15(3):100–8. doi: 10.1007/BF03085963 17612668 PMC1847757

[35] Marasco S. Total Arterial Revascularization. Oper Tech Thorac Cardiovasc Surg. 2016;21(1):20–30. doi: 10.1053/j.optechstcvs.2016.08.002

[36] Diodato M, Chedrawy EG. Coronary Artery Bypass Graft Surgery: The Past, Present, and Future of Myocardial Revascularisation. Surg Res Pract. 2014;2014:1–6. doi: 10.1155/2014/726158 25374960 PMC4208586

[37] Wu J, Hu C, Tang Z, Yu Q, Liu X, Chen H. Tissue-engineered Vascular Grafts: Balance of the Four Major Requirements. Colloids Interface Sci Commun. 2018;23(December 2017):34–44. doi: 10.1016/j.colcom.2018.01.005

