# Supplementary figures and images for: Evaluation of perfusion-driven cell seeding of small diameter engineered tissue vascular grafts with a custom-designed seed-and-culture bioreactor
Source: PLoS One. 2022 Jun 16;17(6):e0269499. doi: 10.1371/journal.pone.0269499 (PMC9202848; doi:10.1371/journal.pone.0269499)

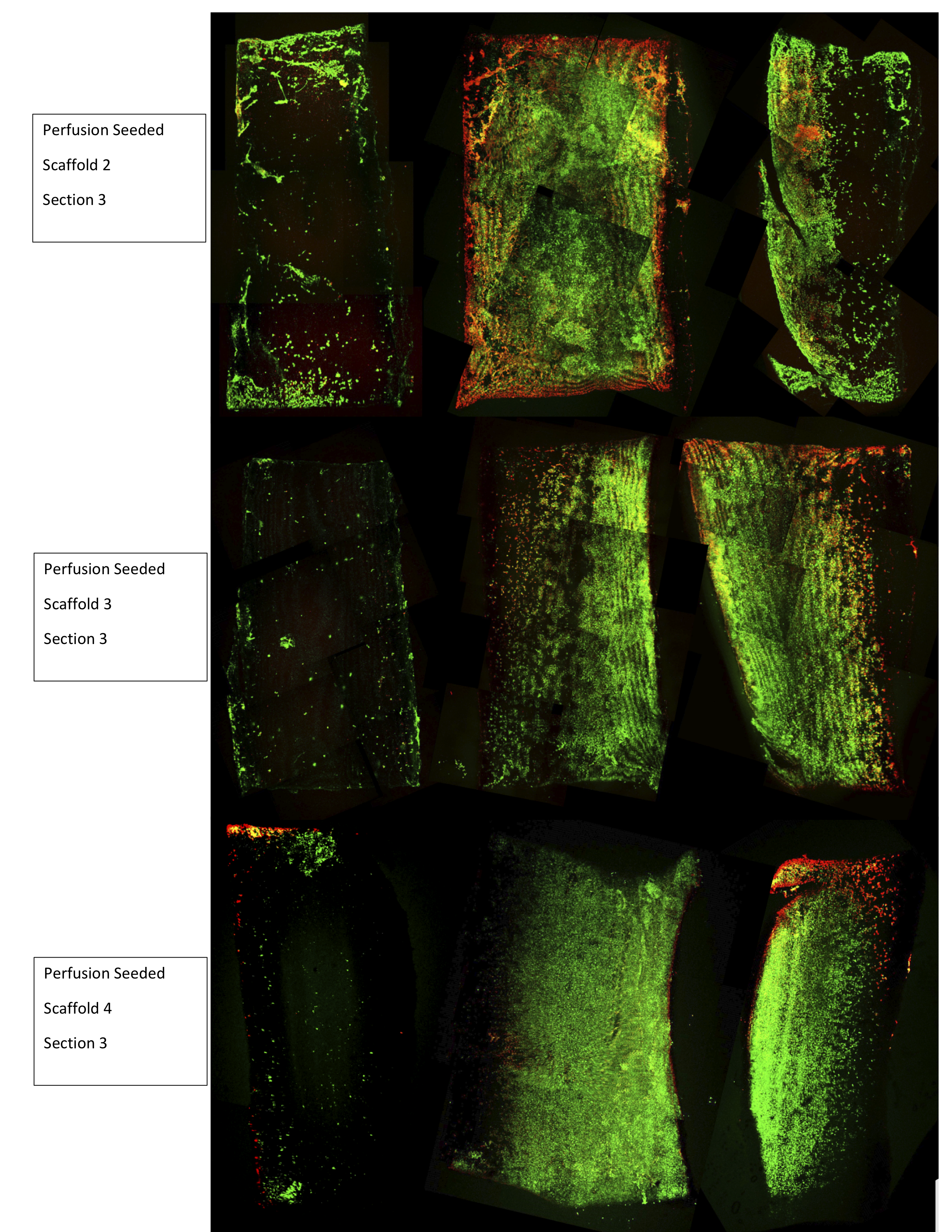

Supplement: S1 Fig — The middle section of the 3 additional perfusion seeded scaffolds are represented in 3rds. Living cells are stained green while dead cells are stained red. View Fig 4 for orientation. (TIFF) [file pone.0269499.s001.tiff]
