# Supplementary material for: Evaluation of perfusion-driven cell seeding of small diameter engineered tissue vascular grafts with a custom-designed seed-and-culture bioreactor
Source: PLoS One. 2022 Jun 16;17(6):e0269499. doi: 10.1371/journal.pone.0269499 (PMC9202848; doi:10.1371/journal.pone.0269499)
Supplement: S1 Table — (PDF) [file pone.0269499.s008.pdf]

		% Circumferential Coverage		Average Cell Layer Thickness		Cell Load	
**Method**	**Section**	**Mean**	**Std Dev**	**Mean**	**Std Dev**	**Mean**	**Std Dev**
Bioreactor Mediated Perfusion	Section 1	66.7	22.7	28.4	10.7	237882.4	54658.7
	Section 2	63.2	22.7	29.2	14.6	230659.8	100137.7
	Section 3	68.2	17.2	24.4	10.8	216369.0	59721.2
	Section 4	64.2	16.4	26.6	13.1	214043.1	53940.6
	Section 5	51.7	29.9	30.3	10.1	204504.7	101938.1
Static Injection Counterpart	Section 1	15.7	18.2	20.3	25.8	102127.5	121275.3
	Section 2	18.7	14.0	22.3	17.3	92039.7	86140.5
	Section 3	21.7	14.9	31.4	25.6	138274.5	107639.5
	Section 4	23.7	17.5	24.4	19.2	126550.0	125010.8
	Section 5	13.7	17.6	17.6	21.7	78319.2	115501.3
Drip	Section 1	49.6	42.0	30.1	15.5	162642.3	60918.8
	Section 2	11.3	10.0	17.5	16.7	47992.6	39424.2
	Section 3	11.6	20.2	11.3	19.6	69727.3	97166.2
	Section 4	0	0	0	0	0	0
	Section 5	15.3	13.2	32.7	37.3	112059.6	127531.7
