# Supplementary material for: Evaluation of perfusion-driven cell seeding of small diameter engineered tissue vascular grafts with a custom-designed seed-and-culture bioreactor
Source: PLoS One. 2022 Jun 16;17(6):e0269499. doi: 10.1371/journal.pone.0269499 (PMC9202848; doi:10.1371/journal.pone.0269499)
Supplement: S2 Table — (PDF) [file pone.0269499.s009.pdf]

	% Area ImageJ	% Area Manual	Error
SIC Seeding	29.1	34.2	3.56
SIC Seeding	0.47	0.66	0.13
SIC Seeding	2.5	4	1.06
SIC Seeding	68.7	70.9	1.54
SIC Seeding	15.8	13.4	1.65
Drip Seeding	61.6	61.5	0.04
Drip Seeding	29.6	31.5	1.4
Drip Seeding	69.7	75.2	3.91
Drip Seeding	5.4	6.5	0.77
Drip Seeding	92.2	94	1.27
BMP Seeding	0.88	0.72	0.11
BMP Seeding	65.4	67.3	1.38
BMP Seeding	35.1	37.6	1.73
BMP Seeding	0.22	0.17	0.03
BMP Seeding	80.4	82.9	1.66
